# Hydrogen Gas Sensing Performances of *p*-Type Mn_3_O_4_ Nanosystems: The Role of Built-in Mn_3_O_4_/Ag and Mn_3_O_4_/SnO_2_ Junctions

**DOI:** 10.3390/nano10030511

**Published:** 2020-03-11

**Authors:** Lorenzo Bigiani, Dario Zappa, Chiara Maccato, Alberto Gasparotto, Cinzia Sada, Elisabetta Comini, Davide Barreca

**Affiliations:** 1 Department of Chemical Sciences, Padova University and INSTM, 35131 Padova, Italy; lorenzo.bigiani@phd.unipd.it (L.B.); alberto.gasparotto@unipd.it (A.G.); 2 Sensor Lab, Department of Information Engineering, Brescia University, 25133 Brescia, Italy; dario.zappa@unibs.it (D.Z.); elisabetta.comini@unibs.it (E.C.); 3 Department of Physics and Astronomy, Padova University and INSTM, 35131 Padova, Italy; cinzia.sada@unipd.it; 4 CNR-ICMATE and INSTM, Department of Chemical Sciences, Padova University, 35131 Padova, Italy; davide.barreca@unipd.it

**Keywords:** Mn_3_O_4_, Ag, SnO_2_, plasma assisted-chemical vapor deposition, hydrogen gas sensors

## Abstract

Among oxide semiconductors, *p*-type Mn_3_O_4_ systems have been exploited in chemo-resistive sensors for various analytes, but their use in the detection of H_2_, an important, though flammable, energy vector, has been scarcely investigated. Herein, we report for the first time on the plasma assisted-chemical vapor deposition (PA-CVD) of Mn_3_O_4_ nanomaterials, and on their on-top functionalization with Ag and SnO_2_ by radio frequency (RF)-sputtering, followed by air annealing. The obtained Mn_3_O_4_-Ag and Mn_3_O_4_-SnO_2_ nanocomposites were characterized by the occurrence of phase-pure tetragonal α-Mn_3_O_4_ (hausmannite) and a controlled Ag and SnO_2_ dispersion. The system functional properties were tested towards H_2_ sensing, yielding detection limits of 18 and 11 ppm for Mn_3_O_4_-Ag and Mn_3_O_4_-SnO_2_ specimens, three orders of magnitude lower than the H_2_ explosion threshold. These performances were accompanied by responses up to 25% to 500 ppm H_2_ at 200 °C, superior to bare Mn_3_O_4_, and good selectivity against CH_4_ and CO_2_ as potential interferents. A rationale for the observed behavior, based upon the concurrence of built-in Schottky (Mn_3_O_4_/Ag) and *p*-*n* junctions (Mn_3_O_4_/SnO_2_), and of a direct chemical interplay between the system components, is proposed to discuss the observed activity enhancement, which paves the way to the development of gas monitoring equipments for safety end-uses.

## 1. Introduction

The reliable detection of hazardous/flammable gases is of key importance in a variety of fields, encompassing disease diagnosis, environmental monitoring and human health protection [1,2,3,4,5,6]. In this broad scenario, a key role is played by the early recognition of molecular hydrogen (H_2_), a zero-emission and clean fuel, with a high energy density of ≈130 MJ×kg^−1^ [2,7], which has emerged as a future energy source for transportation, industrial and residential applications [8,9,10]. Nevertheless, since H_2_ is colorless, odorless and highly flammable, the detection of hydrogen leakages at concentrations lower than hazardous levels [11,12,13,14] is extremely critical towards the emergence of a future hydrogen economy [7,8,15,16,17,18,19].

Simple architecture, cost-effective fabrication, stability under the operating conditions, and high efficiency, are the main requirements and core features of advanced sensors needed for such applications [16]. Among the various active systems and devices [20,21,22,23,24], metal oxide nanostructures have been the subject of an increasing interest, thanks to their high carrier mobility, easy fabrication and excellent stability [9,25,26,27,28]. In particular, whereas *n*-type oxide semiconductors have been largely investigated as gas sensors [8,9,12,15], *p*-type ones have not yet been widely studied [4,17,29,30], since their responses are typically lower than those of *n*-type systems with comparable morphology [31,32,33]. Nonetheless, *p*-type oxide semiconductors have an important potential as gas sensors, and represent promising platforms for the development of devices exhibiting new functions [32], taking into account their appreciable activity as oxidation catalysts and the possibility of boosting their performances by tailoring their chemico-physical properties [2,31,34,35].

Among *p*-type systems, Mn_3_O_4_ has received significant attention due to its low cost, large natural abundance, environmentally friendly character and versatile chemico-physical properties, including the coexistence of mixed valence states [36,37]. Over the last decade, different studies have reported on Mn_3_O_4_-containing gas sensors for various analytes, including CH_3_CH_2_OH, CH_3_COCH_3_, NH_3_ and chemical warfare agent simulants [31,35,36,37,38,39,40,41]. Nonetheless, only two works on Mn_3_O_4_-based gas sensors for molecular hydrogen detection are available in the literature so far [35,42], and the implementation of H_2_ sensors endowed with improved sensitivity and selectivity undoubtedly requires additional research efforts [29,35,37,38].

Beside tailoring the system morphology [25,26,33,39], a proficient way to enhance the functionality of bare Mn_3_O_4_ gas sensors involves their sensitization with suitable metal/oxide agents [8,18,35,42,43,44]. The ultimate aim of this strategy is the exploitation of synergistical chemical and electronic effects, in order to obtain improved performances at moderate working temperatures, an issue of key importance for the development of low power consumption devices [4,37,41]. In this context, the present study is devoted to the fabrication of Mn_3_O_4_-based chemo-resistive sensors for H_2_ detection, sensitized through the on-top deposition of selected metal and metal oxide activators. As prototypes for the two categories, in this work our attention has been focused on the use of Ag, a potential catalyst promoting the reactions involved in the sensing process [45,46,47,48,49], and of SnO_2_, by far one of the most investigated metal oxides for gas sensing applications, endowed with high electron mobility and gas sensitivity [10,13,25,26,50]. In particular, the occurrence of Schottky (Mn_3_O_4_/Ag) or *p*-*n* (Mn_3_O_4_/SnO_2_) junctions between the system components can indeed enhance the modulations of the space charge region, and of the measured electrical resistance, ultimately yielding improved sensing performances thanks to electronic effects [18,26,33,43]. 

**Figure 1:**
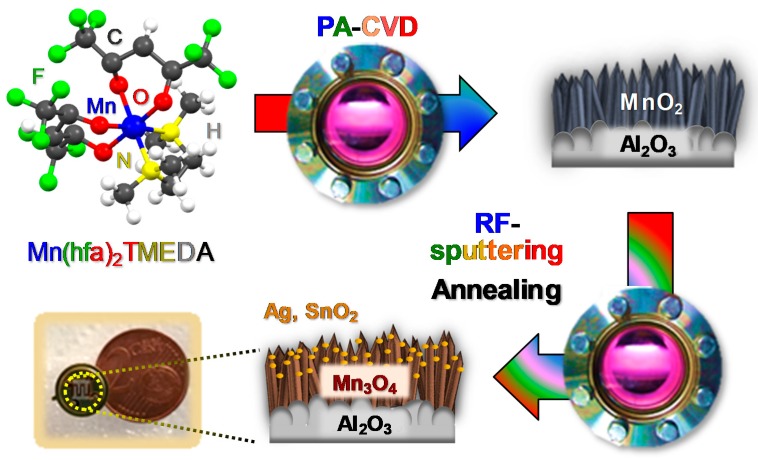
Scheme of the route proposed in the present study for the fabrication of Mn_3_O_4_-Ag and Mn_3_O_4_-SnO_2_ nanomaterials.

At variance with our previous studies, which have involved the fabrication of Mn_3_O_4_-based sensors by means of thermally activated chemical vapor deposition (CVD)-based processes [31,38,39,41], in this work a novel two-step plasma-assisted route was adopted for the preparation of the present materials. The fabrication procedure (Figure 1) involved: (i) the initial plasma assisted-CVD (PA-CVD) on alumina substrates of MnO_2_ from Mn(hfa)_2_TMEDA (Hhfa = 1,1,1,5,5,5-hexafluoro-2,4-pentanedione; TMEDA = *N*,*N*,*N’*,*N’*-tetramethylethylenediamine) [51,52], a molecular precursor never utilized so far for PA-CVD processes; (ii) the functionalization with Ag or SnO_2_ by means of radio frequency (RF)-sputtering, and (iii) a final thermal treatment in air to trigger the transformation of MnO_2_ into Mn_3_O_4_. The main focus of the present investigation was directed at elucidating the structural, compositional and morphological characteristics of the target materials and their interplay with the resulting sensing performances in hydrogen detection. The latter were investigated at a fixed humidity level as a function of the operating temperature, with particular regard to the role exerted by the formation of metal-oxide (Mn_3_O_4_/Ag) or oxide-oxide (Mn_3_O_4_/SnO_2_) junctions. The obtained results indicate that the proposed preparation method yields H_2_ sensors exhibiting favorable detection limits, promising responses at moderate temperature, as well as selectivity against carbon dioxide and methane as potential interferents. To the best of our knowledge, this is the first report on hydrogen gas sensing by Mn_3_O_4_-based composites prepared by a plasma-assisted route.

## 2. Experimental Procedure

### 2.1. Material Preparation

MnO_2_ nanomaterials were initially deposited using a two-electrode plasmo-chemical instrumentation [53] equipped with a RF-generator (*ν* = 13.56 MHz), using Mn(hfa)_2_TMEDA [51,52] as the manganese molecular source. Depositions were performed on pre-cleaned [15,31,39] polycrystalline Al_2_O_3_ substrates (99.6%, Maruwa, Owariasahi, Japan; thickness = 0.25 mm), mounted on the grounded electrode, from Ar-O_2_ plasmas, using the following pre-optimized experimental settings: RF-power = 20 W; growth temperature = 300 °C; total pressure = 1.0 mbar). In a typical process, the precursor powders (weight = 0.15 g) were placed in an external glass reservoir heated at 70 °C, and their vapors were transported into the reactor using an electronic grade Ar flow (60 standard cubic centimeters per minute (sccm)) through gas lines maintained at 130 °C by means of external heating tapes. Additional Ar and O_2_ flows (15 and 5 sccm, respectively) were separately introduced into the reactor. 

Deposition of Ag or SnO_2_ over the obtained systems was subsequently performed through RF-sputtering experiments, carried out using the above described instrumentation, and utilizing silver (Alfa Aesar^®^, Ward Hill, MA, USA; thickness = 0.3 mm, purity ≥ 99.95%) or tin targets (Neyco^®^, Vanves, France; thickness = 2.0 mm, purity = 99.99%). In each experiment, the used target and the Al_2_O_3_-supported manganese oxide deposits were mounted on the RF and grounded electrode, respectively. Depositions were carried out using an Ar flow rate of 10 sccm (total pressure = 0.3 mbar), a RF-power of 5 W, and a grounded electrode temperature of 60 °C. After a preliminary optimization, the deposition time was set at 45 and 90 min for silver and tin sputtering, respectively.

After preparation, ex-situ thermal treatments in air at a temperature of 400 °C for 1 h were carried out in order to ensure the conversion of MnO_2_ into phase-pure Mn_3_O_4_ [54] and to stabilize the obtained nanomaterials in view of gas sensing tests [19,48].

### 2.2. Material Characterization

X-ray diffraction (XRD) patterns were recorded at room temperature using a Bruker (Billerica, MA, USA) D8 Advance X-ray diffractometer with a Cu Kα X-ray source (*λ* = 1.54 Å) operated at 40 kV and 40 mA, employing an incidence angle of 1.0°. The average crystallite dimensions were calculated by means of the Scherrer equation [29,33,36,55]. 

X-ray photoelectron spectroscopy (XPS) analysis was carried out by means of a Perkin-Elmer (Chanhassen, MN, USA) Φ 5600ci instrument equipped with a hemispherical electron analyzer, using a standard Al Kα X-ray excitation source (*hν* = 1486.6 eV). The charging effect on the measured binding energies (BEs) was corrected by adjusting the position of the adventitious C1s signal to 284.8 eV [56]. Atomic percentage (at.%; uncertainty = ±2%) values were determined by peak integration using Φ V5.4A sensitivity factors, after a Shirley-type background subtraction. Silver and tin molar fractions were calculated as *X*_M_ = [(M at.%)/(M at.% + Mn at.%) × 100], with M = Ag, Sn [38,46]. Spectra were fitted with mixed Gaussian–Lorentzian peak shapes using the XPSPEAK program [57]. Auger parameters *α*_1_ and *α*_2_ for silver were calculated according to the literature [56]. A Cameca (Gennevilliers CEDEX, France) IMS 4f spectrometer was used for in-depth secondary ion mass spectrometry (SIMS) analyses, performed at pressures lower than 1 × 10^−10^ mbar, using a Cs^+^ primary beam (14.5 keV, 20 nA, stability 0.2%) and negative secondary ion detection. Depth profiles were acquired using an electron gun for charge compensation in beam blanking mode and high mass resolution configuration. Elemental signals were recorded rastering over a 150 × 150 μm^2^ area and sampling secondary ions from an 8 × 8 μm^2^ region to avoid crater effects. The erosion time in the abscissa of the recorded profiles was converted into depth, based on thickness values measured by field emission-scanning electron microscopy (FE-SEM) measurements. The latter were carried out on a Zeiss (Oberkochen, Germany) SUPRA 40VP instrument at operating voltages of 5.00 kV. Sample thickness and nanoaggregate size values were obtained by analyzing cross-sectional and plane-view images with the ImageJ^®^ software [58]. Atomic force microscopy (AFM) measurements were performed under normal air conditions in tapping mode, using a NT-MDT (Moscow, Russia) SPM Solver P47H-PRO apparatus. Root-mean-square (RMS) surface roughness values were obtained from 3 × 3 μm^2^ micrographs after plane fitting.

### 2.3. Gas Sensing Tests

To avoid any influence from the external environmental conditions, the used gas sensing test system is composed of a stainless-steel chamber located inside a temperature-stabilized climatic chamber, which was set at 20 °C for all measurements. The relative humidity level inside the stainless-steel chamber was constantly monitored and controlled to be exactly 40% at 20 °C. As the sensing devices are completely sealed inside the test chamber (dark conditions), there is no effect from external room illumination. Gas sensing properties of the fabricated systems were investigated using the flow-through method at atmospheric pressure. A constant synthetic air flow (300 sccm) was used as a carrier for the dispersion of H_2_ (and of CH_4_ and CO_2_ in the selectivity tests) at the desired concentrations. Pt interdigitated contacts and a Pt heater were deposited by sputtering (applied power = 30 W, Ar plasma, room temperature) on the active material surface and on the alumina substrate backside (lateral dimensions = 3 × 3 mm^2^), respectively. Resistance values were obtained by applying a bias voltage of 0.2 V, measuring the flowing current through a picoammeter. The measurements were performed in the 100–300 °C temperature interval, after pre-stabilization for 8 h at each temperature to reach a steady state. In line with our previous studies on Mn_3_O_4_-based gas sensors [31,38,39,41], no appreciable resistance variations upon gas exposure were obtained for working temperatures <150 °C, whereas the use of temperatures higher than 300 °C was intentionally avoided in order to prevent Mn_3_O_4_ thermal alterations during sensing tests.

No significant baseline resistance drift was detected after testing up to 12 h. The sensor response was calculated from the measured values of equilibrium resistances in air (*R*_A_), and in the presence of the target gas (*R*_G_), using the following relation [7,31,38,43]:

(1) $$\mathrm{Response}\;=\;\left(\frac{R_{G}-R_{A}}{R_{A}}\right)\times 100$$

A detailed comparison of the present results with previous literature studies was performed by converting the reported response values into those defined by Equation (1). Repeated measurements under the same operating conditions on up to 10 identical sensors yielded stable and reproducible responses (maximum uncertainty = ±10%). The same variation was also estimated to be the response drift upon repeated tests up to 4 months, highlighting thus the system stability, an important issue in view of eventual real-world end uses [37,38,48]. 

The experimental response vs. concentration trends were fitted by the typical power law relation for metal oxide sensors [9,30,42]:

(2) $$\mathrm{Response}\;=\;a\times C^{b}$$

where *C* is the gas concentration, whereas *a* and *b* are constants dependent on the active material and the stoichiometry of the involved reactions [8,31,44]. Detection limits (maximum estimated uncertainty = ±1 ppm) were extrapolated at a response value of 3, assuming the validity of Equation (2) at low analyte concentrations.

## 3. Results and Discussion

### 3.1. Chemico-Physical Characterization

The fabrication process of the target materials is illustrated in Figure 1. Particular efforts were dedicated to elucidating the interplay between the adopted processing conditions and material chemical, physical and functional properties.

**Figure 2:**
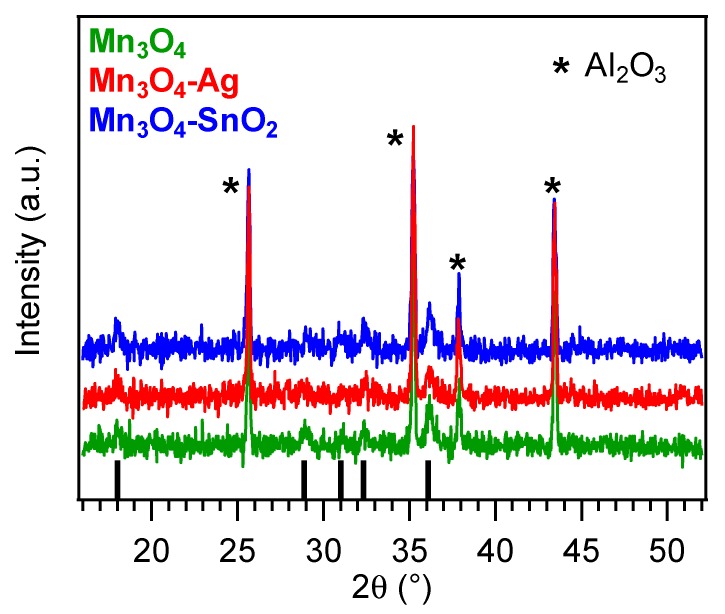
X-ray diffraction (XRD patterns for bare and functionalized Mn_3_O_4_ nanosystems. Vertical black bars correspond to α-Mn_3_O_4_ signals [59].

The system structure was investigated by XRD analyses (Figure 2). All the observed reflections located at 2*θ* = 18.0°, 28.9°, 31.0°, 32.3° and 36.1° could be indexed to the (101), (112), (200), (103) and (211) planes of tetragonal hausmannite (α-Mn_3_O_4_; *a* = 5.762 Å and *c* = 9.470 Å [31,38,59]). The occurrence of relatively weak and broad diffraction peaks suggested the formation of defective nanocrystallites [11], whose average dimensions were close to 25 nm for all the target specimens. A comparison of the signal relative intensities with those of the reference pattern [59] did not reveal any significant orientation/texturing effect, and no appreciable reflections from other Mn oxide polymorphs could be distinguished, highlighting the occurrence of phase-pure systems. Upon functionalization of Mn_3_O_4_ by RF-sputtering, no net variation in the recorded XRD patterns took place. The absence of noticeable diffraction peaks related to Ag or SnO_2_ was traced back to their low content and high dispersion into the Mn_3_O_4_ systems [19,41,44,46] (see also XPS and SIMS results). 

**Figure 3:**
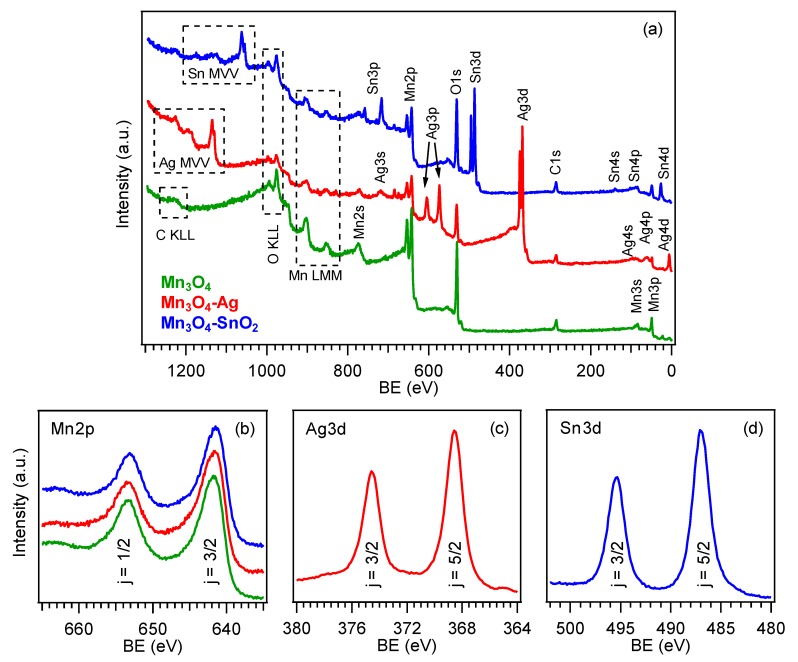
(**a**) X-ray photoelectron spectroscopy (XPS) wide-scan spectra pertaining to bare Mn_3_O_4_, Mn_3_O_4_-Ag and Mn_3_O_4_-SnO_2_ samples. (**b**) Mn2p, (**c**) Ag3d and (**d**) Sn3d photoelectron peaks. The color code is reported in panel (**a**).

The surface chemical state of the developed materials was analyzed by means of XPS. Figure 3a displays the survey spectra for the target specimens, that revealed the presence of oxygen, manganese and eventually, silver or tin signals, for the functionalized systems, together with a minor carbon contribution (<10 at.%) resulting from adventitious contamination. The detection of manganese signals even after RF-sputtering suggested only a partial coverage of Mn_3_O_4_ by the deposited silver- and tin-containing species. Accordingly, Ag and Sn molar fractions were evaluated to be 47.0% and 31.0%, respectively. For bare Mn_3_O_4_, the Mn2p_3/2_ component was located at BE = 641.8 eV (spin-orbit splitting (SOS) = 11.5 eV, Figure 3b), in accordance with previous literature data [31,35,38,41]. For the functionalized systems, a lower Mn2p_3/2_ BE was observed (641.7 eV, for Mn_3_O_4_-Ag, and 641.5 eV, for Mn_3_O_4_-SnO_2_). This finding suggested the formation of Schottky and *p*-*n* junctions for Mn_3_O_4_-Ag and Mn_3_O_4_-SnO_2_, respectively [33,37,38,41,42], resulting in an Ag → Mn_3_O_4_ and SnO_2_ → Mn_3_O_4_ electron transfer. This phenomenon, more pronounced for SnO_2_-containing samples, as testified by the higher BE decrease, exerted a favorable influence on the resulting gas sensing performances. As regards silver (Figure 3c), the Ag3d_5/2_ position (BE = 368.5 eV, SOS = 6.0 eV), as well as the pertaining Auger parameters (see also Figure S1; *α*_1_ = 719.7 eV and *α*_2_ = 725.6 eV), revealed a partial Ag surface oxidation, i.e., the coexistence of Ag(0) and Ag(I) oxide, as typically observed in similar cases [38,46,49,60]. Finally, the main tin photopeak (Figure 3d; BE(Sn3d_5/2_) = 486.9 eV; SOS = 8.4 eV) was located at higher energies than those reported for SnO_2_ [14,56,61], in line with the above mentioned charge transfer process. Taken together, these results highlighted the formation of nanocomposites in which the single components maintained their chemical identity, and enabled us to discard the formation of ternary phases, in line with XRD results. The deconvolution of O1s photopeaks (Figure S2) revealed the concurrence of two distinct bands at BE = 530.0 eV, resulting from lattice oxygen in Mn_3_O_4_, Ag(I) oxide (Mn_3_O_4_-Ag) or SnO_2_ (Mn_3_O_4_-SnO_2_) [14,31,38,60,61], and 531.6 eV, assigned to oxygen species adsorbed on surface O defects [4,41,51,52,55]. The contribution of the latter component to the overall O1s signal increased from ≈36.0%, for bare Mn_3_O_4_, to ≈58.0%, for the functionalized specimens, indicating a parallel increase of the oxygen defect content. The latter feature had a direct beneficial impact on the resulting gas sensing behavior.

**Figure 4:**
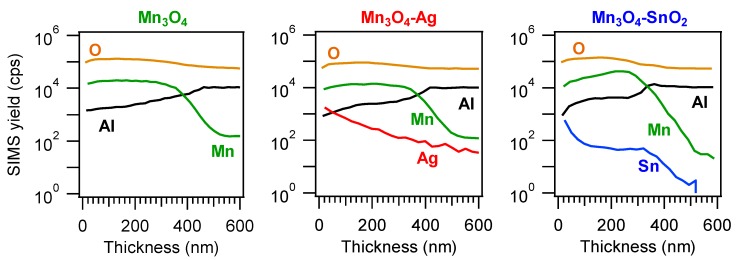
Secondary ion mass spectrometry (SIMS) depth profiles for Mn_3_O_4_, Mn_3_O_4_-Ag and Mn_3_O_4_-SnO_2_ specimens.

**Figure 5:**
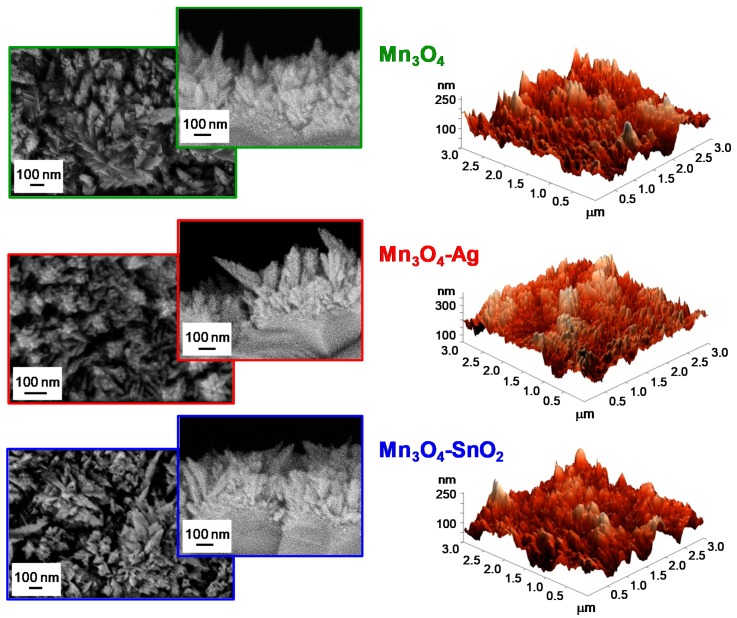
Representative plane-view and cross-sectional field emission-scanning electron microscopy (FE-SEM) micrographs (left panels) and atomic force microscopy (AFM) images (right panels) for Mn_3_O_4_, Mn_3_O_4_-Ag and Mn_3_O_4_-SnO_2_ samples.

Complementary information on material chemical composition was obtained by SIMS in-depth profiling (Figure 4). Upon functionalization of Mn_3_O_4_, no significant variations in the overall deposit thickness took place (for all specimens, the average value was (400 ± 50) nm, as determined by cross-sectional FE-SEM analyses (see below and Figure 5)). The almost parallel trends of manganese and oxygen ionic yields suggested their common chemical origin, in line with the formation of phase-pure Mn_3_O_4_. Silver and tin trends could be described by an erfchian profile, such as in thermal diffusion processes [49]. For the Mn_3_O_4_-SnO_2_ sample, Sn yield underwent a progressive decrease throughout the outer 100 nm, subsequently followed by a plateau, whereas, for the Mn_3_O_4_-Ag specimen, the silver curve continuously declined even at higher depth values. In spite of these differences, a penetration of both Ag and Sn up to the interface with the alumina substrate was observed, and ascribed to the synergistical combination between the inherent RF-sputtering infiltration power and the Mn_3_O_4_ deposit open morphology [38,41,44,48] (see also Figure 5). This intimate contact between the system components is indeed an issue of key importance in order to benefit from their mutual electronic interplay, as discussed in detail below.

The system morphology was investigated by the complementary use of FE-SEM and AFM. FE-SEM micrographs (see Figure 5, left side) highlighted that bare Mn_3_O_4_ was characterized by the presence of elongated nanoaggregates (mean size = 100 nm), whose interconnection resulted in the formation of arrays with an open morphology. This feature is indeed favorable in view of gas sensing applications, since a higher area available for the interaction with the surrounding gases has a beneficial effect on the ultimate material functional performances [15,19,25,31,40]. After Ag and SnO_2_ introduction, no marked variations involving aggregate coalescence/collapse could be observed, validating the potential of the adopted synthetic route in functionalizing Mn_3_O_4_ nano-deposits without any undesired morphological alteration. AFM analyses (Figure 5, right side) confirmed the presence of the aforementioned aggregates uniformly protruding from the growth substrate, resulting in a crack-free and homogeneous granular topography, yielding an average RMS surface roughness of 40 nm for all the analyzed specimens.

### 3.2. Gas Sensing Performances

**Figure 6:**
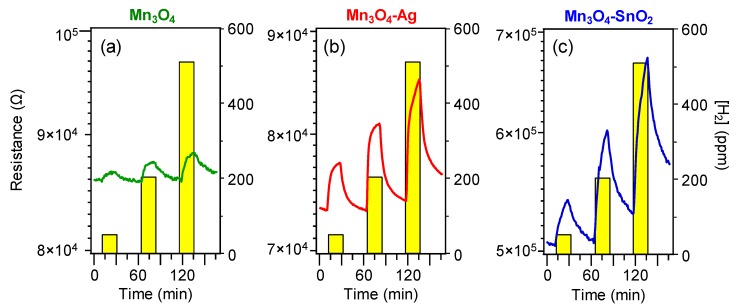
Dynamical responses of Mn_3_O_4_ (**a**), Mn_3_O_4_-Ag (**b**) and Mn_3_O_4_-SnO_2_ (**c**) nanosystems vs. different H_2_ concentrations, at a fixed working temperature of 200 °C.

Figure 6 displays representative dynamical responses of the developed sensors towards square concentration pulses of gaseous hydrogen. All the target materials exhibited a *p*-type sensing behavior, as indicated by the resistance increase upon H_2_ exposure due to the reaction of the analyte with adsorbed oxygen species, resulting in a decrease of the major *p*-type carrier concentration [2,16,31,35,39]. This phenomenon is in agreement with the fact that Mn_3_O_4_ is the main system component, as indicated by structural and compositional characterization [44].

Remarkably, data in Figure 6 evidence that the on-top deposition of Ag and SnO_2_ was an effective mean to increase the electrical property modulation upon H_2_ exposure with respect to pure Mn_3_O_4_. For both composite systems, the measured resistance underwent a relatively sharp rise upon hydrogen exposure, and a subsequent slower increase up to the end of each gas pulse. This phenomenon suggested that the rate-limiting step in the resistance change was the chemisorption of molecular hydrogen on the sensor surface [15,31,41,48]. In spite of an incomplete baseline recovery after switching off hydrogen pulses, the measured resistance variations were almost proportional to the used hydrogen concentrations, enabling us to rule out significant saturation effects, an important starting point for eventual practical applications [15,38,39].

To account for the performance increase yielded by composite systems, it is necessary to consider the mechanism of hydrogen detection by the target *p*-type materials, which can be described as follows. Upon air exposure prior to contact with the target analyte, oxygen molecules undergo chemisorption processes, yielding the formation of various species [6,9,29,36,43,55], among which O^−^ is the prevailing one in the present working temperature interval [14,17,30,33]:

O_2 (g)_ ⇄ 2O^−^_(ads)_ + 2*h*^+^ (3)

**Figure 7:**
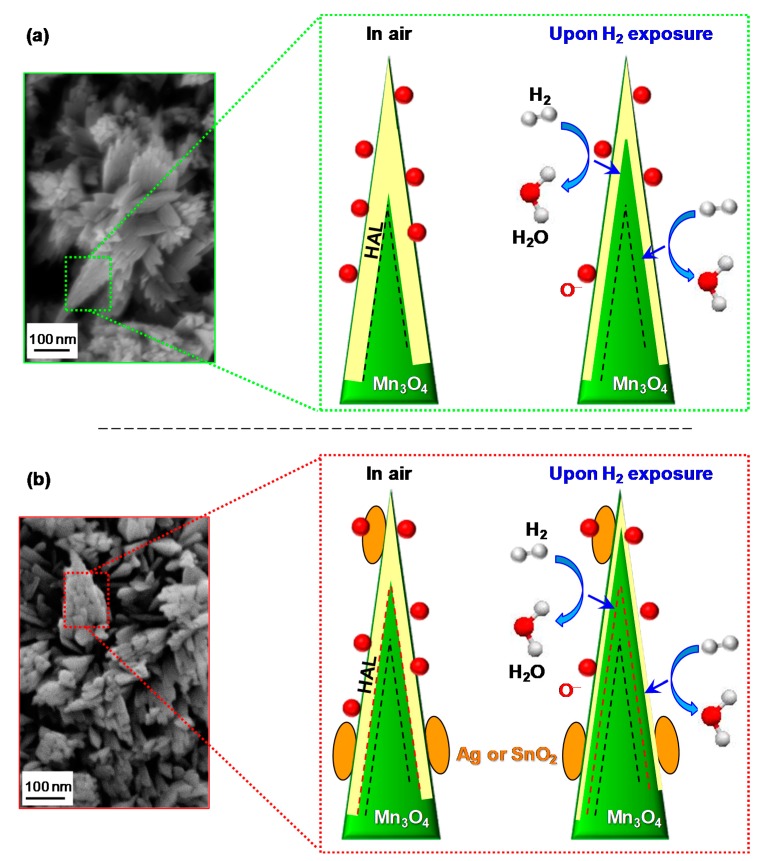
Schematics of the hydrogen gas sensing mechanism and corresponding hole accumulation layer (HAL) modulation for nanosystems based on: (**a**) bare Mn_3_O_4_; (**b**) functionalized Mn_3_O_4_. The dashed black and red lines indicate the HAL boundaries in air in case of bare and functionalized Mn_3_O_4_, respectively. Red spheres, yellow areas, and orange ovals indicate adsorbed oxygen, HAL thickness, and functionalizing agent (Ag or SnO_2_), respectively. Blue arrows indicate electron flow due to H_2_ oxidation (see reaction 4).

As a consequence, the formation of a low resistance hole accumulation layer (HAL) in the near surface Mn_3_O_4_ region takes place (Figure 7; HAL thickness = 20.6 nm, see the Supporting Information) [32,33,37,47]. The subsequent analyte chemisorption is accompanied by electron injection into the system conduction band [3,4,7,29,40,44,62]:

H_2 (ads)_ + O^−^_(ads)_ ⇄ H_2_O _(g)_ + *e*^−^ (4)

A process which decreases the hole concentration and the HAL thickness, resulting, in turn, in an increase of the measured resistance [5,16,17,18,42,43]. Finally, upon switching off the gas pulse, the sensor surface is again in contact with air, and the original situation is restored, with a recovery of the pristine HAL width [30,31,41]. 

Since all the investigated systems have almost identical mean crystallite dimensions, grain size and RMS roughness values, a significant influence of these parameters on the different gas sensing performances can be reasonably ruled out. Indeed, the enhanced responses of composite sensors with respect to the pristine Mn_3_O_4_ can be first explained in terms of electronic effects occurring at the interface between Mn_3_O_4_ and the functionalizing agents, a key aspect to be considered for a deep understanding of gas sensing phenomena [26].

For Mn_3_O_4_-Ag sensors, these processes result from the formation of Mn_3_O_4_/Ag Schottky junctions, whose occurrence produces a Ag → Mn_3_O_4_ electron flow [38,63] (see also the above XPS data), and a consequent thinning of the HAL width in comparison with bare Mn_3_O_4_ (compare Figure 7a,b). As a consequence, HAL variations upon contact of the sensor with gaseous H_2_ produce higher responses by increasing the registered resistance modulations [38,44]. An analogous phenomenon occurs for Mn_3_O_4_-SnO_2_ systems (Figure 7b; HAL thickness = 12.4 nm, see the Supporting Information), although in this case the SnO_2_ → Mn_3_O_4_ electron flow is triggered by a different phenomenon, i.e., the presence of *p*-*n* Mn_3_O_4_/SnO_2_ junctions [32,33,37,41,42,43,55].

The latter effect can, in principle, result in enhanced variations of the HAL extension with respect to the case of Mn_3_O_4_-Ag sensors, since the occurrence of a partial silver oxidation (as evidenced by XPS analysis, see above) precludes a full exploitation of electron transfer effects resulting from the establishment of Mn_3_O_4_/Ag Schottky junctions [38,46]. 

Nonetheless, the enhanced hydrogen detection efficiency of Mn_3_O_4_-based nanocomposites with respect to bare manganese oxide is likely due to the concurrence of additional cooperative phenomena. For both Mn_3_O_4_-Ag and Mn_3_O_4_-SnO_2_ systems, the higher content of oxygen defects at the composite surface with respect to bare Mn_3_O_4_ (see the above XPS data and Figure S2), as well as the exposure of a high density of heterointerfaces, can in fact supply active sites for a more efficient chemisorption of both oxygen and analyte molecules, which, in turn, boosts the resulting gas responses [4,8,16,17,18,30,43]. In addition, the intimate component contact enabled by the adopted preparation route, yielding a good intergranular coupling, enables a proficient exploitation of their chemical interplay [38,44,48], related to the synergistical combination of materials with different catalytic activities [10,27,37,41,47]. Hence, the improved sensing performances of functionalized Mn_3_O_4_ systems can be related to the concomitance of electronic and catalytic effects.

**Figure 8:**
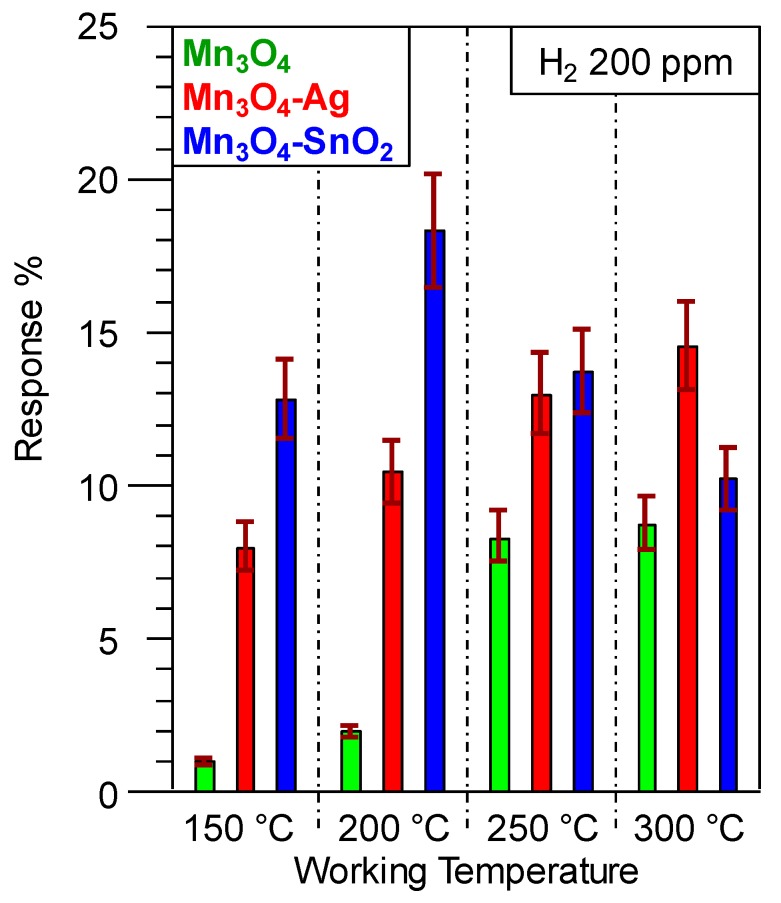
Gas responses to a fixed H_2_ concentration (200 ppm) for the target Mn_3_O_4_-based sensors at different working temperatures.

Taken together, the above observations can account for the improved performances at lower working temperatures of SnO_2_-containing systems with respect to Ag-containing ones. This result is exemplified by an inspection of Figure 8, showing that, apart from the appreciable response enhancement, deposition of Ag and SnO_2_ onto Mn_3_O_4_ resulted in different trends as a function of the operating temperature. In the case of Mn_3_O_4_-Ag sensors, the progressive response rise indicated an enhanced extent of reaction (4) upon increasing the thermal energy supply [2,8,15,31,38,39]. Conversely, as concerns Mn_3_O_4_-SnO_2_, a maximum-like response behavior was observed, the best operating temperature being 200 °C. Such a response trend, in line with previous reports regarding H_2_ detection by other metal oxides [7,9,17,19,29,43,64], suggested the occurrence of a steady equilibrium between hydrogen adsorption and desorption at 200 °C, whereas an increase of the working temperature resulted in a predominant analyte desorption [27,33,55,64,65]. The lower value of the optimum operating temperature for Mn_3_O_4_-SnO_2_ in comparison to Mn_3_O_4_-Ag is in line with the more efficient SnO_2_ → Mn_3_O_4_ electron transfer (see the above XPS data).

The potential of the present results is highlighted by the fact that the best H_2_ responses obtained for Mn_3_O_4_-Ag (at 300 °C) and Mn_3_O_4_-SnO_2_ (at 200 °C) were higher than those reported for sensors based not only on Mn_3_O_4_ [35,42], but even on other *p*-type oxides, including CuO [3,29], NiO [2,30], BiFeO_3_ [7], Co_3_O_4_ [16,19], Ni*_x_*Co_3-*x*_O_4_ [16], as well as nanocomposites based on CuO-Pt [4] and CuO-WO_3_ [18]. In addition, the same responses compared favorably with those pertaining to various MnO_2_-based nanomaterials/thin films in the detection of the same analyte [1,64,65]. A comparison of selected representative data is reported in Table S1. It is also worthwhile highlighting that the optimal operating temperature for H_2_ detection by the present materials (200 °C for Mn_3_O_4_-SnO_2_ systems) was lower than the ones reported for Mn_3_O_4_ [35], MnO_2_ [8], CuO [3,17,29], Co_3_O_4_ [16], NiO [9], NiO-ZnO [43], Ni*_x_*Co_3-*x*_O_4_ [16], BiFeO_3_ [7] and CuO-WO_3_ sensors [18]. This result is of importance, not only to avoid dangerous temperature-triggered explosions, but also to implement sensing devices with a higher service life and a lower power consumption [11,25,38,41].

Gas responses were also analyzed as a function of H_2_ concentration (Figure S3). The obtained linear trends in the log-log scale confirmed the absence of appreciable saturation phenomena, an important prerequisite for a quantitative analyte detection [37,38,39,44,48]. The best detection limits obtained by fitting experimental data with equation (2) ((18 ± 1) ppm and (11 ± 1) ppm for Mn_3_O_4_-Ag and Mn_3_O_4_-SnO_2_ sensors, respectively) were close to those previously reported for MnO_2_ [8], CoO [6] and CuO-TiO_2_-Au [44] sensors, and inferior than those pertaining to ZnO ones [15]. It is also worth noticing that these values were nearly three orders of magnitude lower than the H_2_ lower explosion limit (LEL, 40000 ppm) [2,11,12,23,43], highlighting thus the detection efficiency of the present systems.

Beyond sensitivity, selectivity is an important parameter for the eventual utilization of gas sensing devices [6,11,19,27,30]. The responses towards a specific test gas are in fact required to be higher than those of other potential interferents, in order to avoid false alarms in real-time gas monitoring equipment [36,38,39]. In particular, the choice of CH_4_ and CO_2_ as potential interferents in real-time hydrogen leak detection is motivated by the fact that: (i) hydrogen and methane are common reducing gases, either stored, or used together [1,13]; (ii) the presence of carbon dioxide may hamper hydrogen recognition [5,21,43] in the case of fuel cells eliminating CO_2_ and producing electricity and H_2_ [66]. As shown in Figure S4, the present sensors yielded no responses towards CO_2_, and only weak signals upon exposure to CH_4_. In the latter case, the selectivity was estimated as the ratio between the responses to H_2_ and CH_4_ [32,41], yielding values of 22 and 24 for Mn_3_O_4_-Ag and Mn_3_O_4_-SnO_2_ at the best working temperatures (300 and 200 °C, respectively). Though preliminary in nature, the latter results are an attractive starting point for further studies aimed at implementing exclusive H_2_ sensors, which are highly required for practical applications [13].

## 4. Conclusions

In summary, in this work we have proposed an unprecedented fabrication route to Mn_3_O_4_-Ag and Mn_3_O_4_-SnO_2_ nanocomposites, consisting in: (i) the PA-CVD of MnO_2_ on alumina substrates; (ii) the subsequent introduction of Ag and SnO_2_, as prototypes of metal and oxide functional activators, by means of RF-sputtering; (iii) final thermal treatment in air. A thorough chemico-physical investigation revealed the formation of high purity nanocomposites, characterized by the presence of phase-pure hausmannite Mn_3_O_4_ featuring a close contact with Ag and SnO_2_. The successful obtainment of Schottky (Mn_3_O_4_/Ag) and *p*-*n* junctions (Mn_3_O_4_/SnO_2_) offered significant benefits in view of gas sensing applications, resulting in a nearly ten-fold enhancement of hydrogen responses in comparison to bare Mn_3_O_4_ (up to 25% to 500 ppm H_2_ at a working temperature as low as 200 °C). This improvement, reinforced by the concurrent chemical interplay between the system components, was accompanied by a good sensitivity (detection limits down to 11 ppm, significantly inferior than the H_2_ LEL of 40,000 ppm) and selectivity in the presence of CH_4_ and CO_2_ as potential interferents. Overall, these issues represent a step forward in the use of *p*-type Mn_3_O_4_-based nanocomposites for an efficient and early recognition of H_2_ leakages in low power consumption sensors.

Future developments of the present activities will concern the extension of the proposed preparation route to different multifunctional materials for (photo)electrochemical water splitting and solar-driven catalysis for air/water purification. In addition, more detailed selectivity studies, including other gaseous species and the evaluation of sensing performances under different environmental conditions, are undoubtedly important issues to be properly considered for the eventual real-world implementation of the developed materials.

## Supplementary Materials

The following are available online at https://www.mdpi.com/2079-4991/10/3/511/s1: Figure S1: Surface silver Auger signal for the Mn_3_O_4_-Ag specimen, Figure S2: Deconvolution of surface O1s XP spectra for Mn_3_O_4_-based samples, Figure S3: Gas responses as a function of H_2_ concentration for bare and functionalized Mn_3_O_4_ sensors, Figure S4: Gas responses to fixed CO_2_, CH_4_, and H_2_ concentrations for Mn_3_O_4_-Ag and Mn_3_O_4_-SnO_2_ sensors, response time data, calculation of the HAL width.
