# Supplementary material for: Hydrogen Gas Sensing Performances of p-Type Mn3O4 Nanosystems: The Role of Built-in Mn3O4/Ag and Mn3O4/SnO2 Junctions
Source: Nanomaterials (Basel). 2020 Mar 11;10(3):511. doi: 10.3390/nano10030511 (PMC7153470; doi:10.3390/nano10030511)
Supplement: Supplementary file 1 [file nanomaterials-10-00511-s001.pdf]

Supporting Material

Hydrogen gas sensing performances of *p*-type Mn_3O_4 nanosystems: the role of built-in Mn_3O_4/Ag and Mn_3O_4/SnO_2 junctions

Lorenzo Bigiani [1], Dario Zappa [2], Chiara Maccato [1,*], Alberto Gasparotto [1], Cinzia Sada [3], Elisabetta Comini [2] and Davide Barreca [4]

[1] Department of Chemical Sciences, Padova University and INSTM, 35131 Padova, Italy; lorenzo.bigiani@phd.unipd.it (L.B.); alberto.gasparotto@unipd.it (A.G.)
[2] Sensor Lab, Department of Information Engineering, Brescia University, 25133 Brescia, Italy; dario.zappa@unibs.it (D.Z.); elisabetta.comini@unibs.it (E.C.)
[3] Department of Physics and Astronomy, Padova University and INSTM, 35131 Padova, Italy; cinzia.sada@unipd.it
[4] CNR-ICMATE and INSTM, Department of Chemical Sciences, Padova University, 35131 Padova, Italy; davide.barreca@unipd.it
* Correspondence: chiara.maccato@unipd.it; Tel.: +39-0498275234

S1. Chemico-physical Characterization

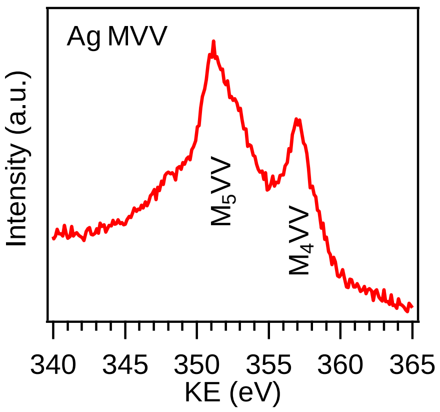

Figure S1. Surface silver Auger signal for the Mn_3O_4-Ag specimen.

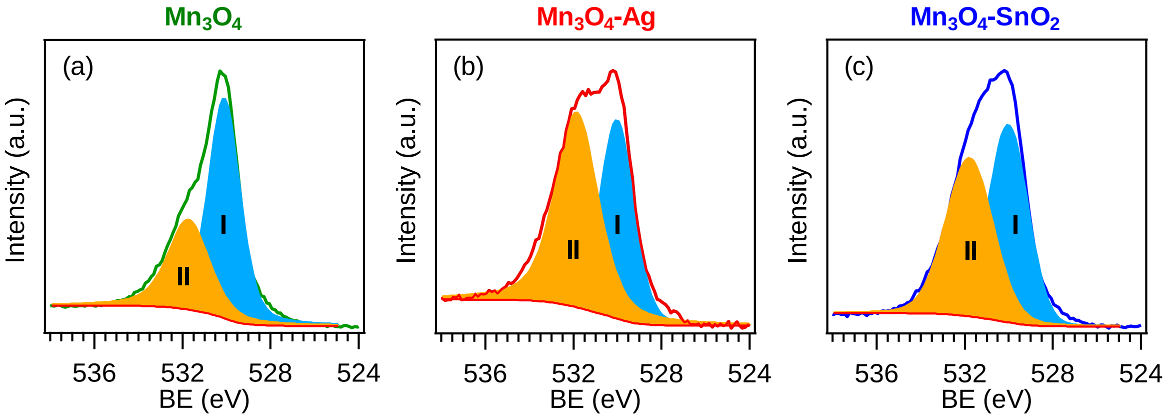

Figure S2. Deconvolution of surface O1s XP spectra for Mn_3O_4 (a), Mn_3O_4-Ag (b), and Mn_3O_4-SnO_2 samples.

S2. Gas Sensing Performances

Response times (τ_{resp}, maximum estimated uncertainty = ±10%) were defined as the times required for the sample resistance to reach 80% of the equilibrium value following hydrogen injection (τ_{resp}) [1-3]. For a hydrogen concentration of 200 ppm at a working temperature of 200°C, typical τ_{resp} values were 360 s, 240 s and 120 s for Mn_3O_4, Mn_3O_4-Ag and Mn_3O_4-SnO_2 specimens, respectively. These results, in line with response data reported in Fig 8 at the same temperature and hydrogen concentration, indicate that functionalization with Ag and SnO_2 produces a faster response with respect to bare Mn_3O_4. The obtained values were lower than the ones previously reported for hydrogen detection by Co_3O_4 [4], NiO [5,6], CuO- [7,8] and MnO_2-based nanosystems [9] under analogous conditions, and comparable to those reported for Pt-Fe_2O_3, Ag/Fe_2O_3 [10] and Ag/ZnO nanomaterials [11]. Nonetheless, the minimization of these values undoubtedly deserves additional research efforts in view of a possible practical use of the developed sensors.

The width of the hole accumulation layer (HAL) in pure p-type Mn_3O_4 can be expressed as follows [12-14]:

$$W_{\mathrm{Mn3O4}} = \left[\frac{2\varepsilon_{\mathrm{Mn3O4}}\Phi}{qN_{\mathrm{Mn3O4}}}\right]^{1/2} \tag{S1}$$

where Φ is the height of the potential barrier established by oxygen adsorption (1.1 eV) [15], ε_{Mn3O4} is the permittivity of Mn_3O_4 ($7.94\times\varepsilon_0$, where ε_0 is the vacuum dielectric permittivity = 8.854×10^{-12} $C^2\times N^{-1}\times m^{-2}$) [16], N_{Mn3O4} is the hole density in Mn_3O_4 (2.25×10^{24} m^{-3}) [17], and q is the electron charge (1.602×10^{-19} C). Using the above values, the calculated width is W_{Mn3O4} = 20.6 nm.

Upon functionalization of Mn_3O_4 systems with SnO_2, the formation of p-n Mn_3O_4/SnO_2 junctions is responsible for the modulation of HAL thickness according to the equation [12,13]:

$$W'_{\mathrm{Mn3O4}} = \left[\frac{2\varepsilon_{\mathrm{Mn3O4}}\varepsilon_{\mathrm{SnO2}}N_{\mathrm{SnO2}}V_0}{qN_{\mathrm{Mn3O4}}(\varepsilon_{\mathrm{Mn3O4}}N_{\mathrm{Mn3O4}} + \varepsilon_{\mathrm{SnO2}}N_{\mathrm{SnO2}})}\right]^{1/2} \tag{S2}$$

Here, V_0 = 0.5 eV is the contact potential difference between SnO_2 and Mn_3O_4, calculated as the difference between the single oxide work function (WF) values [18,19], whereas ε_{SnO2} ($18.2\times\varepsilon_0$) and N_{SnO2} (3.6×10^{24} m^{-3}) are the permittivity and the electron concentration in SnO_2, respectively [20]. The calculation yields W'_{Mn3O4} = 12.4 nm.

In the case of Mn_3O_4-Ag systems, the occurrence of a finite metal/semiconductor junction [21], as well as the partial Ag oxidation (as demonstrated by XPS analyses, see the main paper text), prevent from a detailed and straightforward numerical calculation.

Sensor	H_2 concentration (ppm)	Temperature (°C)	Response $\left(\frac{R_G - R_A}{R_A}\right) \times 100$	Ref.
Mn_3O_4-Ag	200	300	15	Present study
Mn_3O_4-SnO_2	200	200	19	Present study
CuO	200	300	10	[22]
NiO	1000	150	≈0	[23]
$BiFeO_3$	500	300	5	[24]
Co_3O_4	200	600	≈0	[25]
$Ni_xCo_{3-x}O_4$	200	600	1	[25]
MnO_2-rGO	500	85	0.4	[26]
MnO_2-MWCNTs	3×10^4	220	7.5	[27]
MnO_2-WO_3	50-200	200	0	[28]

Table S1. Comparison of hydrogen sensing properties of the present Mn_3O_4-based sensors with selected representative literature works. rGO = reduced graphene oxide; MWCNTs = multi-walled carbon nanotubes.

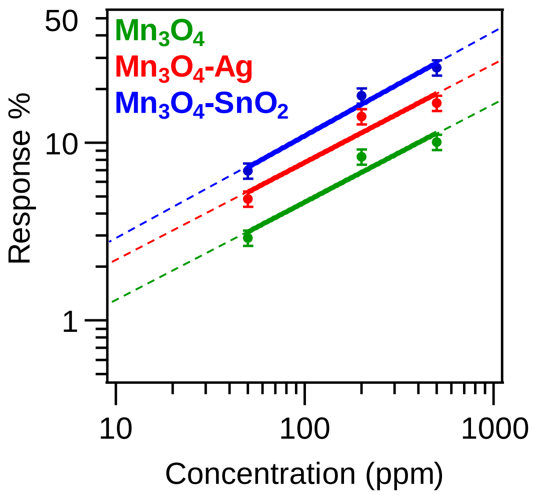

Figure S3. Gas responses as a function of H_2 concentration for bare and functionalized Mn_3O_4 sensors. Working temperature = 200°C for Mn_3O_4-SnO_2; 300°C for Mn_3O_4 and Mn_3O_4-Ag.

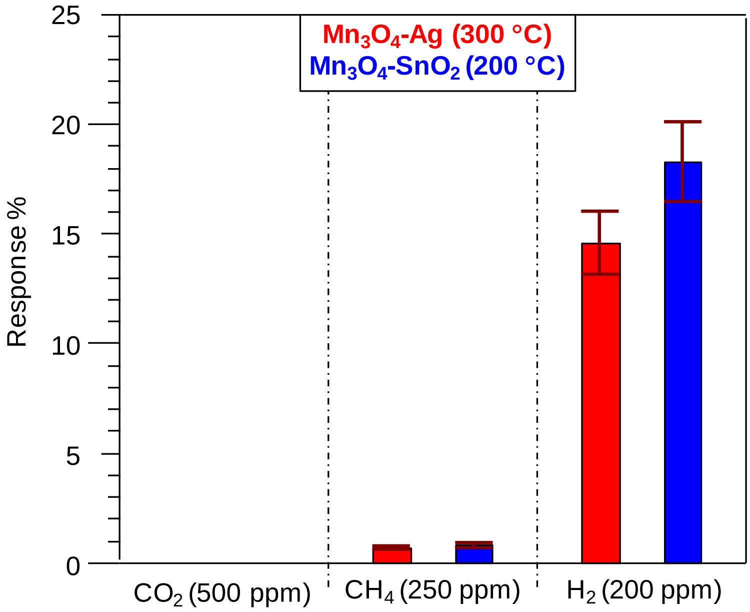

Figure S4. Gas responses to fixed CO_2, CH_4, and H_2 concentrations (500 ppm, 250 ppm and 200 ppm, respectively) for Mn_3O_4-Ag and Mn_3O_4-SnO_2 sensors.

References

1. Bigiani, L.; Zappa, D.; Barreca, D.; Gasparotto, A.; Sada, C.; Tabacchi, G.; Fois, E.; Comini, E.; Maccato, C. Sensing nitrogen mustard gas simulant at the ppb scale via selective dual-site activation at Au/Mn_3O_4 interfaces. *ACS Appl. Mater. Interfaces* **2019**, *11*, 23692–23700.
2. Maccato, C.; Bigiani, L.; Carraro, G.; Gasparotto, A.; Sada, C.; Comini, E.; Barreca, D. Toward the detection of poisonous chemicals and warfare agents by functional Mn_3O_4 nanosystems. *ACS Appl. Mater. Interfaces* **2018**, *10*, 12305–12310.
3. Comini, E.; Baratto, C.; Concina, I.; Faglia, G.; Falasconi, M.; Ferroni, M.; Galstyan, V.; Gobbi, E.; Ponzoni, A.; Vomiero, A., et al. Metal oxide nanoscience and nanotechnology for chemical sensors. *Sens. Actuators, B* **2013**, *179*, 3–20.
4. Barreca, D.; Comini, E.; Gasparotto, A.; Maccato, C.; Pozza, A.; Sada, C.; Sberveglieri, G.; Tondello, E. Vapor phase synthesis, characterization and gas sensing performances of Co_3O_4 and Au/Co_3O_4 nanosystems. *J. Nanosci. Nanotechnol.* **2010**, *10*, 8054–8061.
5. Stamataki, M.; Tsamakis, D.; Brilis, N.; Fasaki, I.; Giannoudakos, A.; Kompitsas, M. Hydrogen gas sensors based on PLD grown NiO thin film structures. *Phys. Status Solidi A* **2008**, *205*, 2064–2068.
6. Kaur, N.; Comini, E.; Zappa, D.; Poli, N.; Sberveglieri, G. Nickel oxide nanowires: vapor liquid solid synthesis and integration into a gas sensing device. *Nanotechnology* **2016**, *27*, 205701.
7. Choi, Y.-H.; Kim, D.-H.; Hong, S.-H.; Hong, K.S. H_2 and C_2H_5OH sensing characteristics of mesoporous *p*-type CuO films prepared via a novel precursor-based ink solution route. *Sens. Actuators, B* **2013**, *178*, 395–403.
8. Sarıca, N.; Alev, O.; Arslan, L.Ç.; Öztürk, Z.Z. Characterization and gas sensing performances of noble metals decorated CuO nanorods. *Thin Solid Films* **2019**, *685*, 321–328.
9. Bigiani, L.; Zappa, D.; Maccato, C.; Comini, E.; Barreca, D.; Gasparotto, A. Quasi-1D MnO_2 nanocomposites as gas sensors for hazardous chemicals. *Appl. Surf. Sci.* **2020**, *512*, 145667.
10. Carraro, G.; Barreca, D.; Comini, E.; Gasparotto, A.; Maccato, C.; Sada, C.; Sberveglieri, G. Controlled synthesis and properties of *β*-Fe_2O_3 nanosystems functionalized with Ag or Pt nanoparticles. *CrystEngComm* **2012**, *14*, 6469–6476.
11. Simon, Q.; Barreca, D.; Bekermann, D.; Gasparotto, A.; Maccato, C.; Comini, E.; Gombac, V.; Fornasiero, P.; Lebedev, O.I.; Turner, S., et al. Plasma-assisted synthesis of Ag/ZnO nanocomposites: First example of photo-induced H_2 production and sensing. *Int. J. Hydrogen Energy* **2011**, *36*, 15527–15537.
12. Choi, S.-W.; Katoch, A.; Kim, J.-H.; Kim, S.S. Remarkable improvement of gas-sensing abilities in *p*-type oxide nanowires by local modification of the hole-accumulation layer. *ACS Appl. Mater. Interf.* **2015**, *7*, 647–652.
13. Sze, S.M.; Ng, K.K. *Physics of semiconductor devices*; John Wiley & Sons, Inc., 3rd Ed.: 2007.
14. Bigiani, L.; Zappa, D.; Maccato, C.; Gasparotto, A.; Sada, C.; Comini, E.; Barreca, D. Mn_3O_4 nanomaterials functionalized with Fe_2O_3 and ZnO: fabrication, characterization, and ammonia sensing properties. *Adv. Mater. Interfaces* **2019**, *6*, 1901239.
15. Larbi, T.; Ouni, B.; Boukhachem, A.; Boubaker, K.; Amlouk, M. Investigation of structural, optical, electrical and dielectric properties of catalytic sprayed hausmannite thin film. *Mater. Res. Bull.* **2014**, *60*, 457-466.
16. Ben Said, L.; Boughalmi, R.; Inoubli, A.; Amlouk, M. Electric conduction mechanisms study within Zr doped Mn_3O_4 hausmannite thin films through an oxidation process in air. *Appl. Microsc.* **2017**, *47*, 131–147.
17. Larbi, T.; Haj Lakhdar, M.; Amara, A.; Ouni, B.; Boukhachem, A.; Mater, A.; Amlouk, M. Nickel content effect on the microstructural, optical and electrical properties of *p*-type Mn_3O_4 sprayed thin films. *J. Alloys Compd.* **2015**, *626*, 93–101.
18. Maniak, G.; Stelmachowski, P.; Zasada, F.; Piskorz, W.; Kotarba, A.; Sojka, Z. Guidelines for optimization of catalytic activity of 3d transition metal oxide catalysts in N_2O decomposition by potassium promotion. *Catal. Today* **2011**, *176*, 369–372.

19. Li, F.; Gao, X.; Wang, R.; Zhang, T.; Lu, G. Study on TiO_2-SnO_2 core-shell heterostructure nanofibers with different work function and its application in gas sensor. *Sens. Actuators, B* **2017**, *248*, 812–819.
20. Choi, S.-W.; Katoch, A.; Kim, J.-H.; Kim, S.S. Striking sensing improvement of n-type oxide nanowires by electronic sensitization based on work function difference. *J. Mater. Chem. C* **2015**, *3*, 1521–1527.
21. Zhang, Z.; Yates, J.T. Band bending in semiconductors: chemical and physical consequences at surfaces and interfaces. *Chem. Rev.* **2012**, *112*, 5520–5551.
22. Duc, L.D.; Le, D.T.T.; Duy, N.V.; Hoa, N.D.; Hieu, N.V. Single crystal cupric oxide nanowires: length- and density-controlled growth and gas-sensing characteristics. *Physica E* **2014**, *58*, 16–23.
23. Zhao, S.; Shen, Y.; Zhou, P.; Zhang, J.; Zhang, W.; Chen, X.; Wei, D.; Fang, P.; Shen, Y. Highly selective NO_2 sensor based on *p*-type nanocrystalline NiO thin films prepared by sol–gel dip coating. *Ceram. Int.* **2018**, *44*, 753–759.
24. Bala, A.; Majumder, S.B.; Dewan, M.; Roy Chaudhuri, A. Hydrogen sensing characteristics of perovskite based calcium doped $BiFeO_3$ thin films. *Int. J. Hydrogen Energy* **2019**, *44*, 18648–18656.
25. Govindhan, M.; Sidhureddy, B.; Chen, A. High-temperature hydrogen gas sensor based on three-dimensional hierarchical-nanostructured nickel–cobalt oxide. *ACS Appl. Nano Mater.* **2018**, *1*, 6005–6014.
26. Zöpfl, A.; Lemberger, M.-M.; König, M.; Ruhl, G.; Matysik, F.-M.; Hirsch, T. Reduced graphene oxide and graphene composite materials for improved gas sensing at low temperature. *Faraday Discuss.* **2014**, *173*, 403–414.
27. Jung, D.; Yoon, Y.; Lee, G.S. Hydrogen sensing characteristics of carbon-nanotube sheet decorated with manganese oxides. *Chem. Phys. Lett.* **2013**, *577*, 96–101.
28. Zhang, C.; Boudiba, A.; Navio, C.; Olivier, M.-G.; Snyders, R.; Debliquy, M. Study of selectivity of NO_2 sensors composed of WO_3 and MnO_2 thin films grown by radio frequency sputtering. *Sens. Actuators, B* **2012**, *161*, 914–922.
